# Data related to inflammation and cholesterol deposition triggered by macrophages exposition to modified LDL

**DOI:** 10.1016/j.dib.2016.05.046

**Published:** 2016-05-27

**Authors:** Juan Toledo, Montserrat Esteve, Mar Grasa, Angelo Ledda, Horacio Garda, José Gulfo, Ivo Díaz Ludovico, Nahuel Ramella, Marina Gonzalez

**Affiliations:** a Department of Nutrition and Food Sciences, Faculty of Biology, University of Barcelona, Barcelona, Spain; b CIBER Obesity and Nutrition, Institute of Health Carlos III, Madrid, Spain; c INIBIOLP-CONICET, Facultad Cs. Médicas, Universidad Nacional de La Plata, La Plata, Argentina

**Keywords:** Low density lipoprotein, Peroxidative damage, Cell viability, Macrophages

## Abstract

This article supports experimental evidence on the time-dependent effect on gene expression related to inflammation and cholesterol deposition in lipid-loaded cells. The cells employed were human monocytes THP1 line transformed into macrophages by treatment with phorbol esters. Macrophages were treated at different times with oxidized low density lipoprotein (Ox-LDL) and then gene expression was measured. We also include data about the different types of oxidized lipoprotein obtained (low, media or high oxidation) for differential exposure with Cu ions. These data include characterization to lipid and protein peroxidative damage and also quantification of cell viability by exposure to native and modified LDL. The present article complements data published in “Decreased OxLDL uptake and cholesterol efflux in THP1 cells elicited by cortisol and by cortisone through 11β-hydroxysteroid dehydrogenase type 1” Ledda et al. (in press) [1].

**Specifications Table**

**Table:** 

Subject area	Biology
More specific subject area	Biochemistry and Molecular Biology
Type of data	Figures
How data was acquired	Images were obtained at transmission electron microscope JEM 1200 EX II (JEOL Ltd., Tokyo, Japan) and then photographed by an Erlangshen camera ES1000W (Model, 785 Gatan Inc., Pleasanton, California, USA)
	Analytical measures were registered in a two-beam spectrophotometer -Cintra-20, Sydney, Australia.
	RT-PCR reactions were run on ABI PRISM 7900 HT detection system (Applied Biosystems)
Data format	Analyzed
Experimental factors	THP1 cells line from ATCC® Number: TIB-202™. LDL as obtained from healthy human donors from Instituto de Hemoterapia de la Provincia de Buenos Aires, Argentina
Experimental features	Cellular RNA was extracted with Trizol according to the technique supplied by the manufacturer and cDNA synthesis was performed employing iScript cDNA Synthesis Kit. Real-Time PCR was carried out using SYBR Green PCR Master Mix on an ABI PRISM 7900 HT detection system using an annealing temperature of 60 °C.
Data source location	La Plata, Argentina and Barcelona, Spain
Data accessibility	Data are with this article

**Value of the data**

• These data show the characterization of different types of oxidized LDL that refers to protein and lipid lipoprotein peroxidation. This information could be considered in studies employing Ox-LDL.
• Data presented in this article also show that the degree of LDL oxidation is directly related to damage and viability cell. This result could be potentially important for further investigations related to the initiation of plaques in atherosclerotic lesions.
• Our data show that the expression of genes related to inflammation and lipid accumulation in human macrophages is time-dependent. These data could be helpful for other researchers in the development of experiments related to atherosclerotic diseases.

## 1 Data

The data presented in this article provide information about the characterization of different types of oxidized LDL as well as how modified LDL alters the gene expression related to inflammation process. Dataset shows the methodology used to obtain different types of oxidized low density lipoproteins (Ox-LDL) in order to produce low (L), medium (M), or high (H) peroxidation degree of LDL. We characterized Ox-LDLs (L, M, and H) using quantitative techniques for lipid determination and protein peroxidation. To perform experiments, we selected medium peroxidation degree LDL (M) based on cell viability. We show data about the time-dependency of gene expression involved in the inflammatory process and cholesterol loading.

## 2 Experimental design, materials and methods

### 2.1 Ox-LDL characterization

We have obtained Ox-LDL employing the Cu^2^ oxidation method (see Ref. [21] in [1]). Native human (N-LDL, 3 mg/ml) in a volume of 15 ml was treated with 0.5 ml copper sulfate (3.6 mg/ml) at a final concentration of 5 μM at 37 °C under gentle agitation for 4, 8, or 12 h in order to produce L, M, or H peroxidation degree of LDL. To stop the peroxidation process, each preparation was treated with a solution of butylhydroxytoluene (BHT) in PBS at a final concentration of 0.1 mM and immediately subjected to dialysis against PBS (50 mM, pH 7.40, changed every 8 h) for 24 h to eliminate BHT and Cu ions. Peroxidative damage infringed to lipid and protein components of the native LDL induced by copper treatment was evaluated by thiobarbituric acid reactive substances (TBARS) [3], conjugated dienes formation [2] and protein carbonyls (PCs) [4].

TBARS was fluorimetrically determined to estimate the extent of lipid peroxidation in homogenates [3]. An aliquot of cellular homogenates (50–100 μL) was reacted with 200 μL of SDS (8.10%, W/V) and 1.5 mL of acetic acid 10% (V/V) (pH 3, 5). Subsequently, 1.5 mL of thiobarbituric acid (TBA 0.8%) and 600 μL of water were incorporated and the mixture was heated in sealed tubes at 95 °C for 60 min. Under these conditions TBARS (mainly malondialdehyde (MDA) generated by lipid peroxidation) reacted with TBA to yield TBA-MDA adducts which were quantified at 515 nm excitation and 553 nm emission. The concentration of the chromophore was calculated from a calibration curve prepared with fresh tetrametoxipropane (TMP) solutions (TMP was purchased from Sigma Chem. Co., Buenos Aires, Argentina).

PCs were determined by the method of Reznick and Packer [4]. Aliquots of cellular homogenates were incubated with dinitro-phenylhydrazine in HCL 2 N at 37 °C in the dark for 30 min. The corresponding hydrazone-derivatives present in the proteins were revealed after the addition of excess NaOH and measured at 505 nm. The concentration of PCs was calculated from a calibration curve prepared with a stock solution of sodium pyruvate (Sigma Chem. Co., Argentina).

TBARS formation was expressed as *m* moles of malondialdehyde (MDA)/mg protein (first bar) and conjugated dienes as optical density units (ODU)/mg protein (second bar) are shown in.

**Fig. 1:**
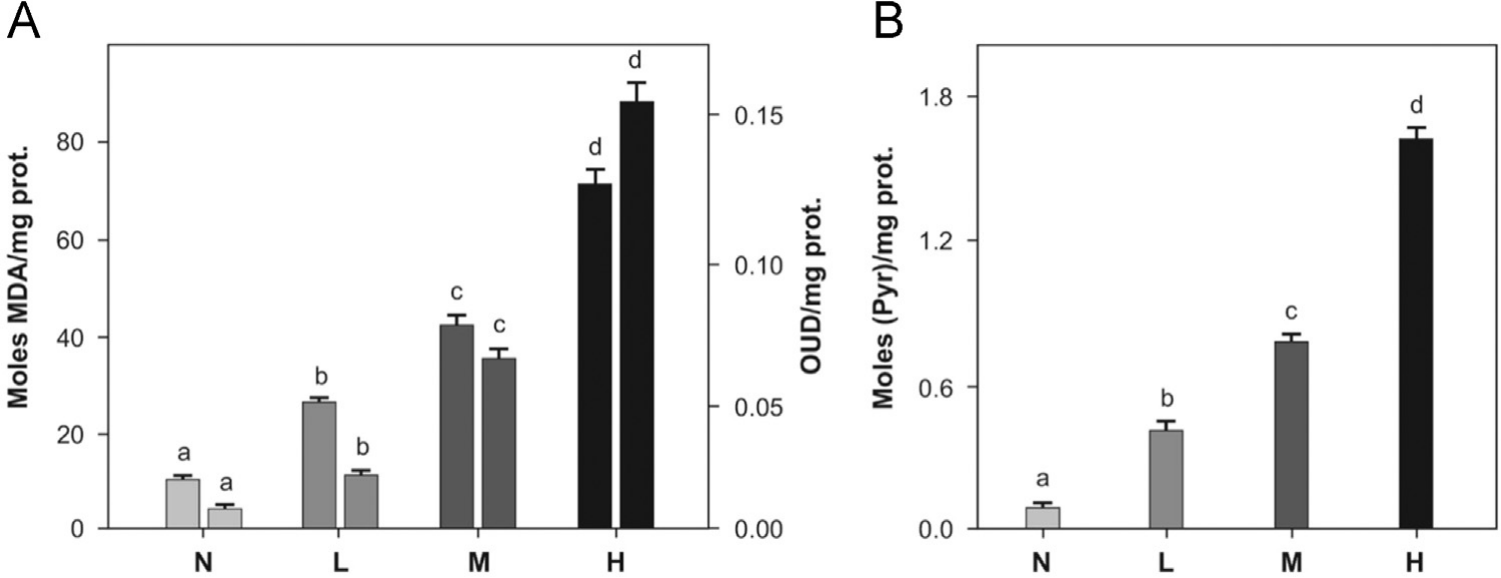
Peroxidative damage infringed to lipid and protein components of the native LDL induced by copper treatment. TBARS formation (A) and carbonylation of aminoacyl residues in LDL protein (B). Each bar is the mean of three independent experiments assayed in triplicate (mean±SD). Statistical differences among data of the same type are indicated with different letters on the top of the bars. Data with distinct letters are statistically different between them at *p*<0.01 signification level (ANOVA+Turkey test).

Fig. 1A Carbonylation of aminoacyl residues in LDL protein after pro-oxidant treatment is shown in Fig. 1B and expressed as *n* moles of pyruvate (Pyr)/mg protein. Degree of peroxidation was denoted by capital letters (N, native LDL; L, light-; M, medium-, and H, high-peroxidized LDL). Each bar represents the mean of three independent experiments assayed in triplicate (mean±SD).

### 2.2 Cytotoxic effect of different types of Ox-LDLs (N, L, M and H)

**Fig. 2:**
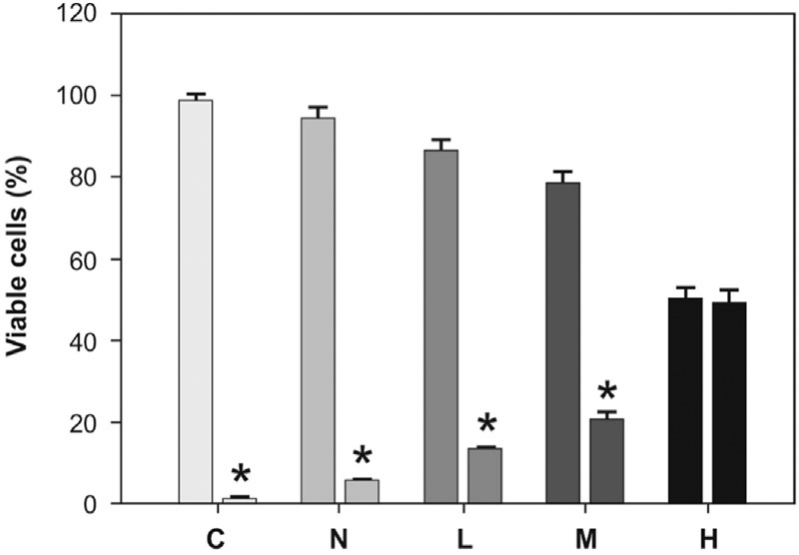
Cell viability by trypan blue dye (TBD) exclusion test in macrophages treated without (C) or with native (N) or different degree of peroxidized LDL (L, M, or H). First bar corresponds to viable cells and second bar to non-viable cells in each condition. *p<0.01 viable respect non-viable cells.

We have evaluated cell viability by trypan blue dye (TBD) exclusion test in macrophages treated with native or peroxidized LDL for 24 h (Fig. 2**)**. The Ox-LDL preparation was diluted 1/10 in culture medium (RPMI). Ox-LDL was added in the culture medium at a final protein concentration of 100 µg/mL at 2 ml final volume for well. According to the evaluation of peroxidation degree of LDL and cells survival test, we have chosen medium (M) peroxidation degree of the LDL to perform experiments described in the present DiB and in the original research article. Briefly, cells were washed with PBS and treated with 100 μL of 0.1% solution of trypan blue dye (in PBS, pH 7.40). After one-min incubation at room temperature (gentle orbital agitation) they were examined under optical microscopy to determine the percentage of viable cells according to the method described by Jauregui et al. [5]. At least four fields of one hundred cells per field were counted and the results were expressed as the percentage of non-viable cells.

### 2.3 Gene expression involved in inflammatory processes

**Fig. 3:**
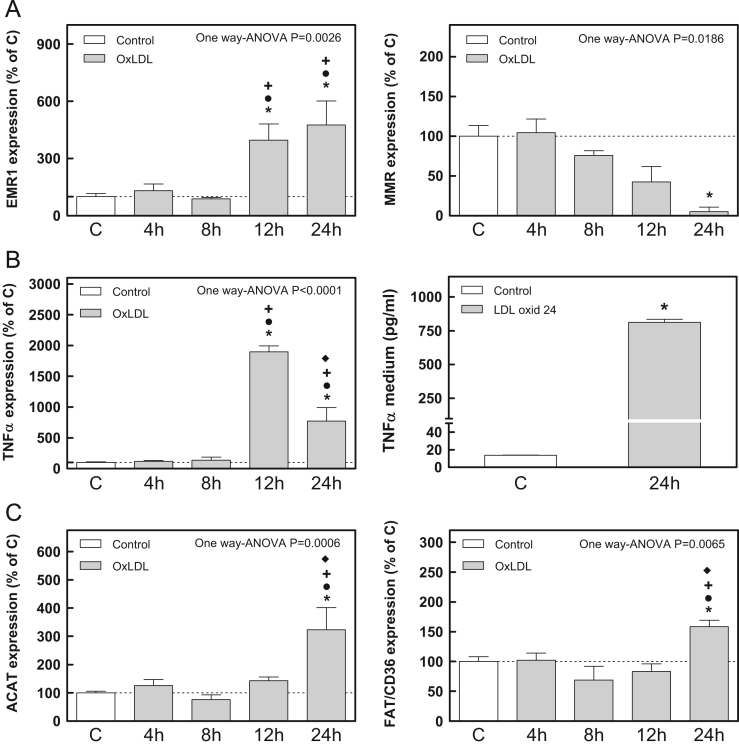
Gene expression related to inflammatory processes in THP1 cells. A. Expression of EMR1 and macrophage mannose receptor (MMR). B Expression of tumor necrosis factor α (TNFα) (left panel) and TNFα secretion (right panel). C. Expression of fatty acid translocase (FAT/CD36) and acyl-CoA:cholesterol acyltransferase (ACAT). Statistical comparisons were performed by one way-ANOVA and Bonferroni post Test analysis: *p<0.05 vs. C; •*p*<0.05 vs. 4h; +*p*<0.05 vs. 8h; ♦*p*<0.05 vs. 12h.

We selected medium (M) peroxidation degree of the LDL to evaluate the expression of genes involved in the inflammatory process. Monocytes humans cells (THP1) were grown in RPMI medium containing 10% of serum fetal bovine (SFB) at 37 °C in a 5% CO_2_ atmosphere. Later, they were stimulated with phorbol esters (PMA-200 nM) for 24 h and transformed into macrophage type. Then, macrophages THP1 were treated with Ox-LDL for 4, 8, 12, and 24 h at a final concentration of 100 µg protein/ml. The expression level of messenger RNA (mRNA) of different genes was quantified by real-time PCR (RT PCR) (see original research article). As a housekeeping gene, the expression of ribosomal protein L4 (RPL4) was measured. To evaluate the inflammation related to the atherogenic process we determined the expression of the genes: epidermal growth factor like module-containing mucin-like hormone receptor-like1 (EMR1), macrophage mannose receptor (MMR) (Fig. 3A) and tumor necrosis factor α (TNFα) (Fig. 3B panel left). To quantify the TNFα secretion in the medium when the cells were treated with Ox-LDL we used a specific enzyme immunoassay from Becton Dickinson Co. (Durham, USA) according to the manufacturer׳s instructions. Captured antibody was Anti-Human TNF-α monoclonal antibody, the detection antibody was Biotinylated anti-Human TNF monoclonal antibody and the enzyme reagent was conjugated with streptavidin. Biotin–streptavidin interaction increased significantly affinity and specificity of the assay (Fig. 3B panel right).

For the study of genes involved in cholesterol influx from macrophages and foam cells formation we analyzed fatty acid translocase (FAT/CD36) and acyl-CoA: cholesterol acyl transferase (ACAT) (Fig. 3C).

### 2.4 Electron microscopy

**Fig. 4:**
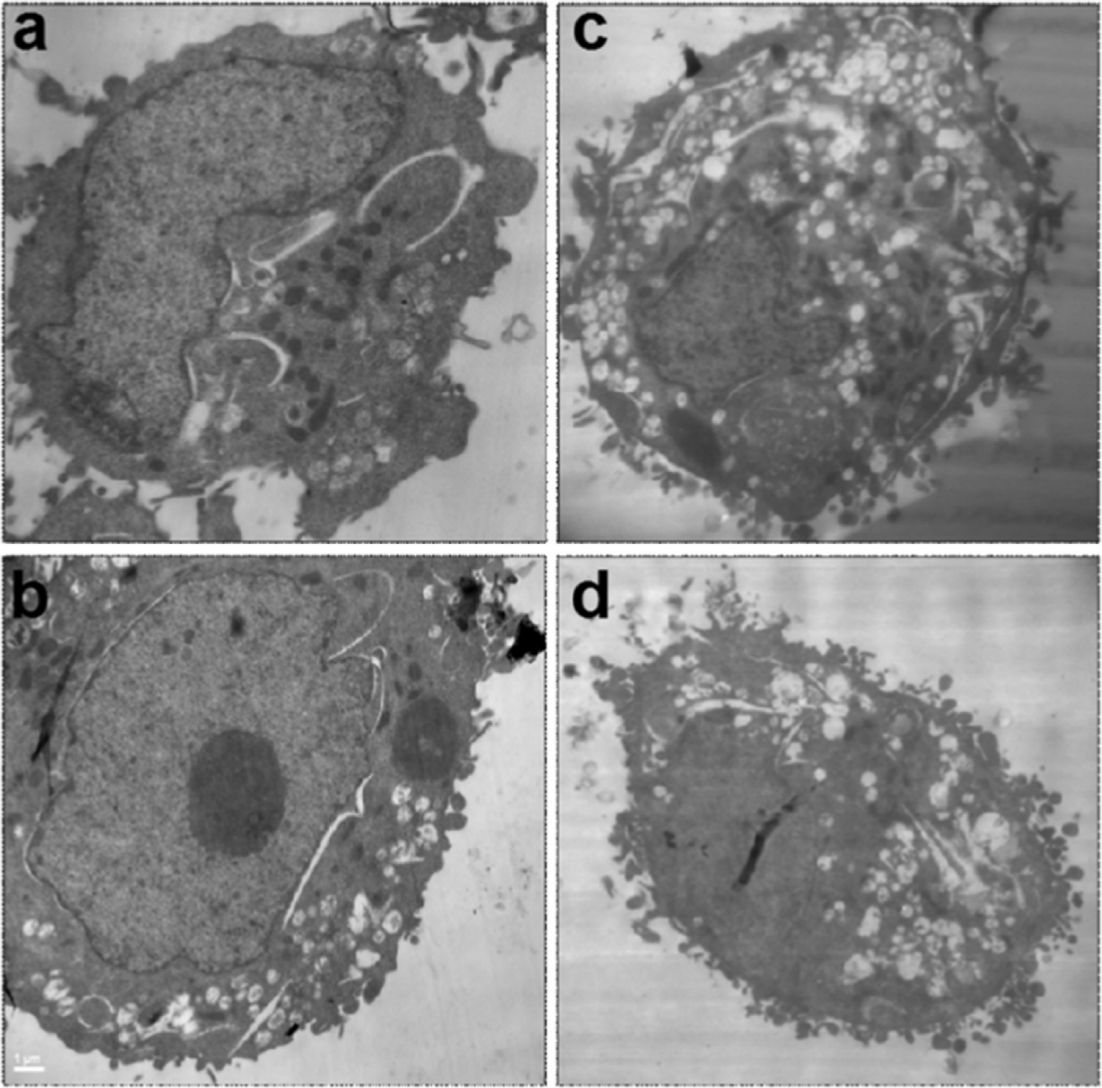
THP cells electron microscopy. Cells were fixed with glutaraldehyde for electron microscopy, a and b were control without Ox-LDL, c and d were treated with Ox-LDL.

The samples treated with or without Ox-LDL were fixed with 2% glutaraldehyde in buffer phosphate (PH 7.2–7.4) for 2 h at 4 °C. Then, cells were centrifuged at 1500 rpm for 10 min. The secondary fixation was performed with osmium tetroxide (1%) for 1 min at 4 °C and then samples were dehydrated with increasing alcohol series and subsequently included in epoxy resin. Ultrathin sections (90 nm) were contrasted with uranyl acetate and lead citrate and examined in a transmission electron microscope JEM 1200 EX II (JEOL Ltd., Tokyo, Japan) and then photographed by an Erlangshen camera ES1000W (Model, 785 Gatan Inc., Pleasanton, California, USA), Central Electron Microscopy service of the Faculty of Veterinary Science, UNLP (Fig. 4).
