# Supplementary material for: Data related to inflammation and cholesterol deposition triggered by macrophages exposition to modified LDL
Source: Data Brief. 2016 May 27;8:251–7. doi: 10.1016/j.dib.2016.05.046 (PMC4906131; doi:10.1016/j.dib.2016.05.046)
Supplement: Supplementary file 1 — Transparency document [file mmc1.docx]

Conflict of interest From

The authors declare that they have no competing interests.
